# Fecal egg counts and individual milk production in temperate pastoral dairy systems of Australia

**DOI:** 10.3168/jdsc.2024-0555

**Published:** 2024-06-28

**Authors:** T. Loughnan, P. Mansell, M. Playford, D. Beggs

**Affiliations:** 1 Colac Veterinary Clinic, Colac, VIC 3250, Australia; 2 Melbourne Veterinary School, Faculty of Veterinary Science, University of Melbourne, Werribee, VIC 3030, Australia; 3 Dawbuts Pty Ltd., Camden, NSW 2570, Australia

## Abstract

Anthelmintic use in lactating dairy cattle has been shown to result in a milk production response in some previous studies. If individual animals within a herd could be identified that would most benefit from anthelmintic treatment, this may reduce anthelmintic resistance. Australian dairy systems are predominantly pasture based, allowing sustained exposure and immune stimulation of cattle to gastrointestinal nematodes. We assessed the relationship between milk production and early-lactation fecal egg counts (FEC) to determine whether cows with higher FEC produced less milk. Ten pasture-based dairy farms in south-west Victoria, Australia, entered an observational study. Individual FEC at a minimum detectable count of 2.5 eggs per gram of feces (epg) were recorded for recently calved primiparous and multiparous cohorts on each farm. All animals were calved ≤30 d at sampling. Body condition scores were assessed at sampling and milk production data were collected from daily milk meters and herd tests to give first 100-d milk production. When separated by primiparous or multiparous status, no difference in the milk production between cows with FEC = 0 and FEC ≥2.5 epg was identified. Between-farm variation was large for FEC and milk production. Fecal egg count at a minimum detectable count of 2.5 epg detected parasitism in primiparous cows postcalving, but the presence and magnitude of parasitism measurable by FEC was not related to milk production in pasture-based Australian dairy systems. In multiparous cows, the rate detection of worm eggs at this analytical sensitivity was lower and the significance of a positive FEC at this analytical sensitivity requires further assessment to ascertain the effect on milk production. Based on our study, it seems unlikely that individual FEC results would be useful as a basis to select individual cows in south-west Victorian dairy herds for anthelmintic treatment.

## Highlights

• We recorded 45% primiparous and 25% multiparous cows with FEC ≥2.5 epg.
• No milk production difference was observed between FEC-positive and -negative cows (2.5 epg analytical sensitivity).
• No milk production differences were observed with higher (≥10 epg) thresholds of FEC cut-off.

## Graphical Abstract

**Figure:**
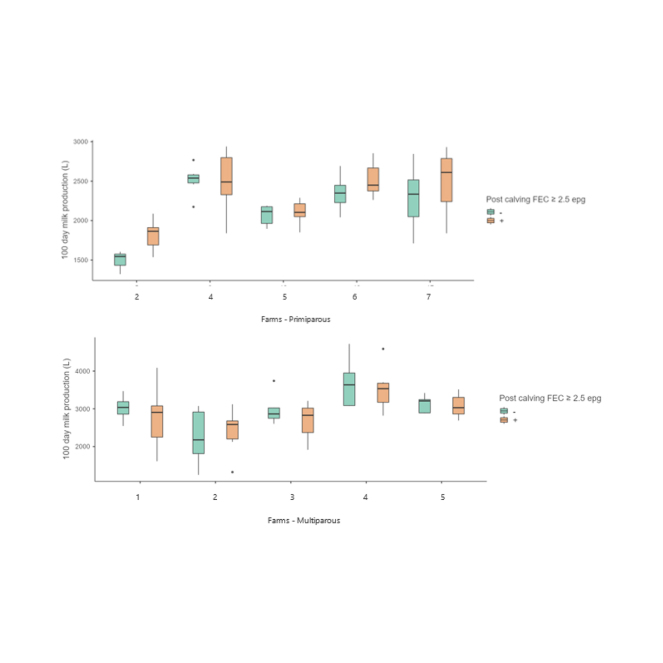
**Summary:** Pasture-based dairy cows were assessed for postpartum fecal egg count (FEC) and 100-day milk production. No relationship was observed between milk production and cows with positive and negative FEC at an analytical FEC sensitivity of 2.5 eggs per gram (epg).

Gastrointestinal nematodes are parasites of cattle that must be managed rather than eradicated. While they can cause clinical disease in young naïve stock (Hilderson et al., 1993), the need for management and treatment in adult dairy cattle has been a contentious topic with varying findings (Charlier et al., 2012; Ravinet et al., 2014; Geurden et al., 2017).

Gastrointestinal nematodes can inflict damage upon abomasal and intestinal wall structures resulting in diarrhea and poor weight gain, but as naïve cattle are exposed to gastrointestinal nematodes, they build resistance and resilience (Hilderson et al., 1993).

As the host immunity against gastrointestinal nematodes develops, the ability for worm egg production is reduced by the host. With stronger immunity, the host cow is able to expel adult worms (Vercruysse and Claerebout, 1997). In a Canadian study, where cattle were at pasture, primiparous cows were 4.5 times more likely to have a detectable fecal egg counts (**FEC**) when compared with multiparous cows (Nødtvedt et al., 2002b). A recent local study, of which this article's data are a subset, illustrated that herds in south-west Victoria have low FEC, and that 45% of primiparous and 26% of multiparous cows have an FEC ≥2.5 epg (Loughnan et al., 2024).

The effect of minor worm burdens on adult dairy cattle and their productivity has been examined repeatedly (Forbes et al., 2004; Mejía et al., 2011; Perri et al., 2011). Local research in south-west Victoria illustrated a 74 L increase in production over the first 100 d of lactation for anthelmintic treated cattle, a 3% milk yield benefit (Walsh et al., 1995). Walsh et al. found no alteration to the milk production response in relation to parity, presence of positive FEC, or BCS. Similar to these findings by Walsh et al. (1995), several international studies in the northern hemisphere have illustrated varied milk production increases among anthelmintic-treated cattle (Nødtvedt et al., 2002a; Ravinet et al., 2014; Verschave et al., 2014). These international studies all include herds with cattle housed for a portion of the year and vary in the approach to timing of treatment; the milk production increase ranged from 0.85 to 0.97 kg/milk per day in these 3 studies.

New Zealand research illustrated an inconsistent milk production response to repeated anthelmintic use across half a herd throughout lactation (Mason et al., 2012). These animals were allowed to comingle and groom after application of a topical anthelmintic. Laffont et al. (2001) illustrated that >60% of topically applied ivermectin achieves eventual fecal excretion because of licking behavior, meaning that selective treatment in comingling cows is likely to have varied responses to therapy.

A meta-analysis of studies investigating the milk production response to anthelmintic treatment was completed in 2004 (Sanchez et al., 2004). Overall, this analysis indicated that 40/75 studies found a positive milk production response associated with anthelmintic use, whereas 34/75 could not establish a relationship between treatment and production. One study in this meta-analysis showed a negative association between treatment and milk production.

In another approach, research in Argentina has illustrated a milk production difference between cows with a positive FEC and those without a positive FEC at an analytical sensitivity of 10 epg (Perri et al., 2011). Cows with a positive FEC at or just before calving produced 7% to 12% less milk than their counterparts with a FEC of 0 epg.

One study in the United Kingdom where anthelmintic was used mid-lactation suggested marginal milk production increase but also an increase in grazing and ruminating time of anthelmintic-treated cows, indicating an increased appetite after parasite removal (Forbes et al., 2004).

Given the inconsistency of findings around treatment responses to anthelmintics in lactating dairy cattle the importance of this topic has greater implications than just the possible liters of milk gained due to treatment. Local trials have shown broad-reaching anthelmintic resistance to all classes of available anthelmintic for dairy and beef cattle (Rendell, 2010; Bullen et al., 2016). Blanket use of anthelmintics in lactating dairy cows is contributing to the development of drug resistance and may be a cost that does not give a return.

We aimed to test the hypothesis that cows with a measurable worm burden, by FEC, would have a lower milk production than their peers without a measurable worm burden at an analytical sensitivity of 2.5 epg of feces.

Ten herds from south-west Victoria, Australia, were enrolled in this study. Herds were selected based on convenience, locality, production system (seasonal or batch calving system to ensure a sufficient number of subjects available), ease of testing due to facilities, and interest from farm to be involved in the study. Eligibility involved being a pasture-based dairy herd in south-west Victoria. Herds with daily milk volume assessments were sought out as study participants to accomplish our aim of production assessment with relation to worm burden. Primarily the herds were selected based on being clients or contacts of the primary researcher.

Herd records from local farms were used to estimate parameters in a power calculation. We considered an increase of 150 L over 100 d to be a biologically important difference. Based on an expected 100-d production of approximately 3,500 L with a standard deviation of 500 L and a positive FEC proportion of 50%, a total of 250 cows would be needed for an 80% power to detect a difference in production of 5%.

All herds were pasture-based grazing operations where cows grazed pasture daily and were supplemented with additional feed in the bale; some farms used feed pads for ration supplementation. Anthelmintics were used on some but not all farms, and date of most recent anthelmintic use was recorded for each cow; the most recent was 59 d before fecal sampling for one individual. This study examined the FEC at a point in time and its relationship with milk production; therefore, anthelmintic use was not considered relevant in this dataset due to time since treatment in all study participants.

Samples were all collected between June 1, 2020, to October 6, 2020. Cows sampled were selected by the farm manager based on age and calving date; all were <30 DIM at the time of sampling. Farm managers selected 15 primiparous cows and 15 multiparous cows, each group of multiparous cows included 3 to 4 animals from each age cohort: 3, 4, 5, and 6+ yr old.

Fecal samples were collected directly from the rectum and transferred into a numbered collection tray. Fecal samples were sent at room temperature via express post to arrive at a commercial laboratory within 48 h.

Cows were condition scored on a scale of 1 to 8 (Dairy Australia, 2013) by the same practitioner at the time of sampling, and identification numbers were recorded to correlate with samples to allow for analysis and later comparison.

Individual animal FEC were assessed down to an analytical sensitivity of 2.5 (epg) using the mini-FLOTAC technology in a ParaBoss-accredited laboratory. The mini-FLOTAC procedure (Cringoli et al., 2017) regularly counts down to an analytical sensitivity of 5 epg; for our study, the mini-FLOTAC procedure was run twice, per sample, to increase the analytical sensitivity to a level of 2.5 epg.

A selection bias occurred to include several herds with daily milk monitors, which resulted in sampling from more rotary herds with greater incorporation of technical equipment. Participation was optional and testing was free to the participant.

During analysis and data cleaning, cows who calved >30 d before date of sampling were excluded from all analyses. Cows were removed from the dataset if they produced less than 500 L in the first 100 d of lactation or did not record a lactation >100 d in length. Three cows recorded production of <500 L over the first 100 d and were deemed to have incomplete datasets. After exclusions, all cohorts consisted of ≥10 cows and the lowest individual production record was 1,235 L.

Milk production data were collected from farms with daily milk volume meters for individual cows (n = 6) and farms where intermittent whole-herd testing was undertaken (n = 4).

Milk production in the first 100 d was calculated in the daily meter farms by summing the milk volumes for d 5 to 100. On intermittent testing farms, d 5 to 100 production was estimated using a weighted average of herd test volumes from cows that had at least one test within 60 d of calving and where there were no more than 80 d between any individual tests.

Statistical analysis was explored using jamovi 1.6.23 (the jamovi project, 2021; retrieved from https://www.jamovi.org). To reduce the effect of farm management, genetics, and nutrition on FEC and production, each animal was ranked on 100-d milk yield and assigned a quartile within their cohort. Graphs showing mean and range of FEC for each quartile were used to observe for relationships between FEC and 100-d yield quartile.

Overall, combining all ages of cattle and all farms into one dataset produced 247 cows with individual FEC and corresponding milk production figures. Herd size ranged from 170 to 1,010 cows with a mean herd size of 551 cows. Data obtained from daily milk meters consisted of 141 individual milk production records and 106 were obtained from extrapolated herd test results. Larval cultures completed from the same sample set used in a parallel study found that 85% of all larvae were *Ostertagia ostertagi* (Loughnan et al., 2024). Of this dataset, 45% of primiparous cows had a FEC ≥2.5 epg, whereas only 25% of multiparous cows had a FEC ≥2.5 epg. Multiparous animals produced on average 3,185 L in their first 100 d of lactation in comparison to 2,255 L for primiparous cows.

Because of the much higher proportion of primiparous cattle with a positive FEC and their lower milk production, the data were separated into primiparous and multiparous sets to avoid interference of the effect of parity on milk production and FEC.

Individual records were obtained for 107 primiparous cows from 8 farms. There was less than a 25 L difference in 100-d milk production between primiparous animals with a FEC ≥2.5 epg and those with an undetectable or 0 FEC (2,244 and 2,268 L, respectively). This was considered to be biologically unimportant. Overall, 140 individual FEC and corresponding milk production records were obtained for multiparous animals across all farms. There was a 115 L difference in the 100-d milk yield between FEC = 0 and FEC ≥2.5 epg groups of multiparous cows as seen in Table 1.

**Table 1:** Comparison of mean 100-d milk production, sample size, and SD for primiparous and multiparous cows with FEC = 0 and FEC ≥2.5 epg

Item	FEC = 0 epg		FEC ≥2.5 epg		*P*-value
	Milk production, L (n)	SD	Milk production, L (n)	SD	
Primiparous	2,244 (59)	499	2,268 (48)	428	0.8
Multiparous	3,218 (100)	585	3,103 (40)	877	0.37

The post hoc decision to analyze primiparous and multiparous cows separately had an impact on the statistical power of our study. Post hoc power calculations suggest that, given the production and variation observed in our results, the statistical power to detect a difference of 150 L over 100 d was only 38% in the primiparous cows and 21% in the multiparous cows. However, the differences we observed were less than 150 L over 100 d in both groups. Thus, while we have no evidence that the production was meaningfully different, we are unable to make further inferences from our data.

A further limitation in calculating the effect of parasitism on milk production in our study was the observed variation in milk production between farms for both primiparous and multiparous cows. To remove the influence of individual farm management on production data and FEC, each animal was ranked among her cohort and placed into quartiles based on individual production level relative to their cohort where 1 was the lowest producing quartile and 4 was the highest. We assumed that if the level of parasitism was important, there should be a difference in the proportion of cows with a positive FEC between the top and bottom quartiles on each farm in terms of milk production. For both multiparous and primiparous cows, the variation in FEC was greatest in the highest producing quartile, but no relationship between FEC and production quartile was observed (Figure 1).

**Figure 1:**
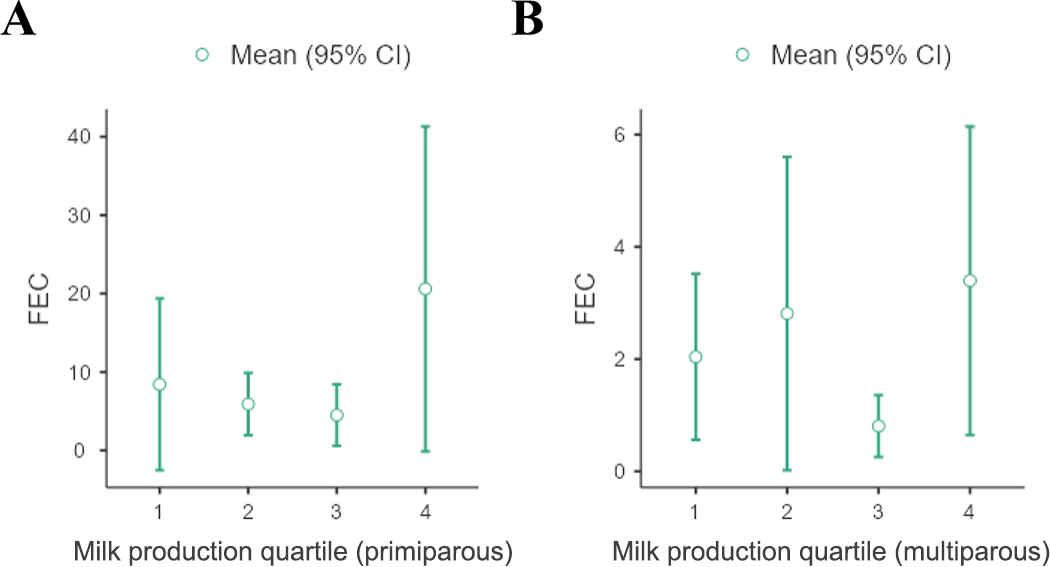
Fecal egg counts with relationship to production quartile for primiparous cows (A) and multiparous cows (B). Individual cows were ranked against their peers within parity and farm groups and assigned to a production quartile; these quartiles were assessed for distribution of FEC. Quartile 4 represented the highest 25% of individual milk yields on each farm.

Assessment of FEC (analytical sensitivity 2.5 epg) and production on a farm-by-farm basis was attempted to reduce the bias of different management, feed, and genetic inputs. For this analysis, some groups were omitted due to low (<30%) proportions of cows with positive FEC. Figure 2A and B show the milk production difference between FEC positive and negative cows where >30% of animals had a FEC ≥2.5 epg.

**Figure 2:**
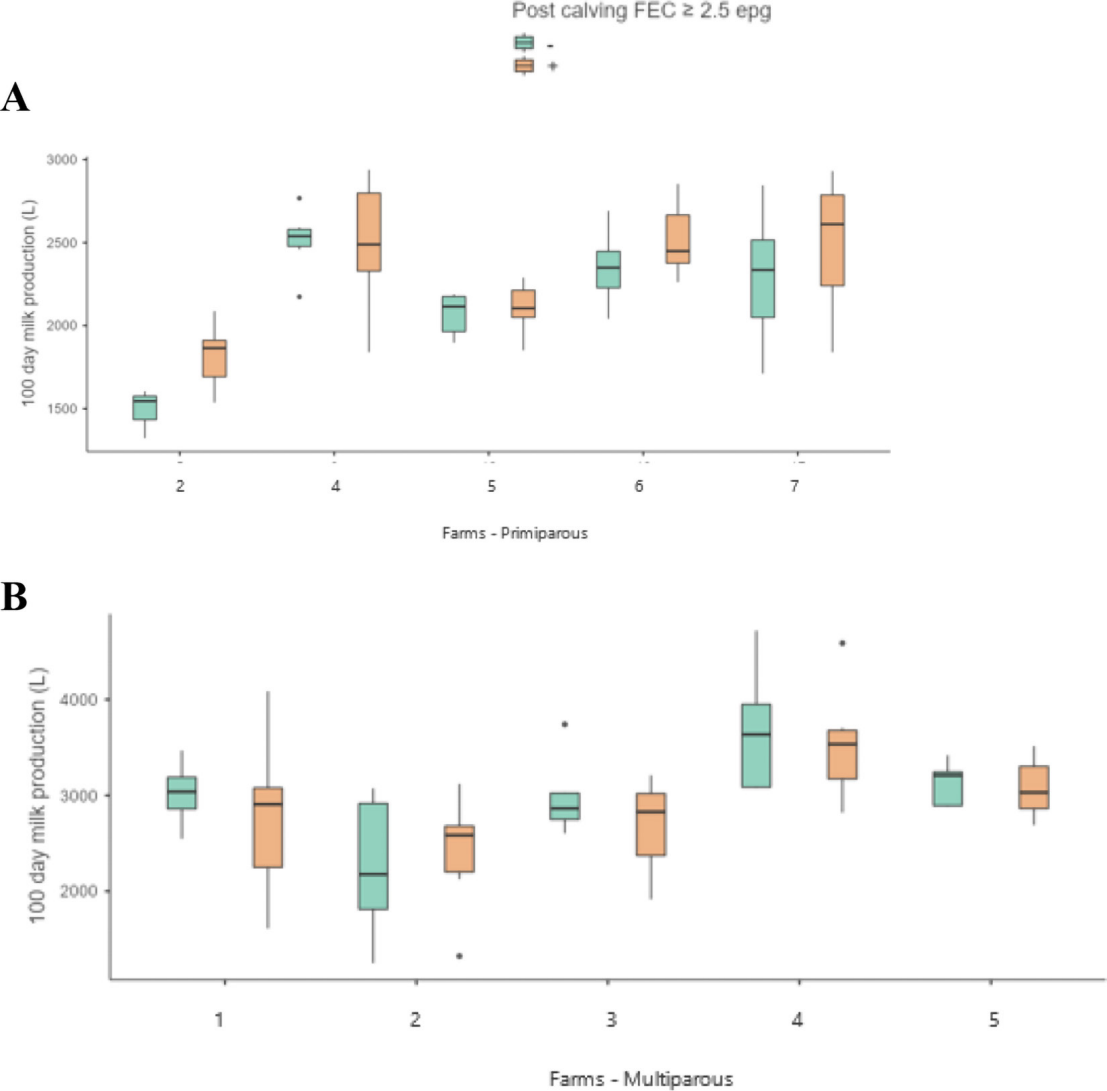
Milk production in cows with a FEC ≥2.5 epg postcalving compared with their peers of FEC = 0 for primiparous cows (A) and multiparous cows (B). Only includes farms where ≥30% of cohort had a positive FEC. Boxplots illustrate the median and interquartile ranges for each group on the farms. The whiskers illustrate the upper and lower quartile ranges with the exception of outliers. Outliers are represented by dots in some plots.

No consistent relationship was observed between milk production and FEC, with some farms having higher milk production in cows with a positive FEC and some farms having lower production in cows with a positive FEC (Figure 2).

We did not observe a relationship between FEC and milk production in primiparous cattle, in contrast to an Argentinian study (Perri et al., 2011) which found that, around calving, the proportion of positive FEC cows was significantly greater in the primiparous group (38% primiparous and 22% multiparous). Unshown data are quoted, stating a significant difference in milk production between FEC positive and negative animals within each parity; however, the overall data shown from their study encompass all parities together (Perri et al., 2011). Overall, our primiparous cows, which had a much higher proportion positive FEC (45% vs. 25% at 2.5 epg), produced 930 L less in their first 100 d.

In contrast to our findings, Perri et al. (2011) found that high-producing cows with an FEC ≥10 epg in the month pre- or postcalving were likely to produce around 7% less milk for the lactation. The level of parasitism evident in our data was not reflective of that found by Perri et al. (2011), who found 22% of multiparous animals had an FEC ≥10 epg around calving; we recorded only 8% of multiparous cows with FEC ≥10 epg.

Walsh et al. (1995) found a milk production response to anthelmintic in all cows; despite this, there was not a difference in milk production response to anthelmintic correlating to FEC, which is aligned with our dataset indicating a lack of correlation between FEC and milk production in primiparous cattle. Contrary to this conclusion, a Belgian study noted a greater milk production response to eprinomectin application at calving in cattle with elevated FEC (Verschave et al., 2014).

Some studies have looked at milk production response to anthelmintics and have compared response to treatment between primiparous and multiparous cows (Verschave et al., 2014; Geurden et al., 2017). Verschave et al. (2014) reported milk production increase by kilograms of milk/cow per day; it may be considered more appropriate to report these production differences as a percentage to remain comparable between parity and regions or systems as primiparous cattle often produce less milk and therefore inherently have a smaller gross change in production. Although Verschave et al. (2014) found a greater milk response to anthelmintic in cows with higher FEC, they found much higher mean FEC in their study herds than was noted in our dataset.

Previously reported production differences in anthelmintic-treated animals may largely be related to appetite rather than feed conversion efficiency as illustrated in a European study (Forbes et al., 2004). Forbes et al. demonstrated that the grazing and ruminating times in anthelmintic treated cows increased by around 50 min (7%) per day, following treatment the ME per kilogram of DM of feces was not different for either anthelmintic-treated or control animals. Much of the previous research indicating a milk production benefit from anthelmintic use did not measure feed intake or grazing time (Nødtvedt et al., 2002a; Ravinet et al., 2014; Verschave et al., 2014).

The choice to use an anthelmintic on lactating dairy cows is not without cost; the most obvious cost is the cost of the product and the labor to apply it. Additional feed costs associated with increased appetite as discussed previously (Forbes et al., 2004) should be considered in any cost-benefit analysis.

Use of anthelmintics in blanket approach for lactating or calving dairy cows to achieve a production response assumes efficacy of product. Previous Australian work (Bullen et al., 2016) has illustrated the diminished efficacy and therefore likely diminished return on investment of the commonly used anthelmintic products for dairy cattle. Interestingly, this study used the injectable anthelmintic treatment in young stock on dairy farms; injectables may have greater efficacy than the pour-on products currently registered for use in Australian lactating dairy cattle. Anthelmintic resistance levels are unknown from the time of previous local work, which illustrated a milk response to anthelmintic (Walsh et al., 1995). It is highly likely that the efficacy of this product has decreased with more than 25 yr of exposure to the same drug across many properties.

Contrary to international practices, Australian young dairy stock are raised on pasture and graze year-round in most operations. International research has shown that previous exposure to gastrointestinal nematodes increases host immunity (Hilderson et al., 1993; Eysker et al., 2000) and is likely to reduce the effect of parasites on growth and production (Ploeger et al., 1990). Furthermore, one study in Canada found that herds with mechanical spreading of feces in pasture where heifers grazed correlated with lower FEC in adult cattle (Nødtvedt et al., 2002b).

Whether or not anthelmintic treatment of dairy cows at a herd level would result in increased production was beyond the scope of our study. We did not see a biologically or economically important production difference in cows with or without a measurable FEC at sensitivities of 2.5 epg. Thus, our results do not suggest that individual FEC results would be useful for selective treatment of individual cows with anthelmintics. Further work is necessary to determine whether there is a basis to reduce blanket treatment of herds with anthelmintics based on individual test results.

However, our work may have implications for future studies. The much greater proportion of primiparous cows with positive FEC compared with multiparous animals (45% vs. 25%) and their lower average milk production (2,255 vs. 3,185 L) required a re-design of the data analysis. The separate analysis of the primiparous and multiparous groups was not planned and was not undertaken in similar studies (Perri et al., 2011; Verschave et al., 2014). One important implication of our study is that future researchers should consider carefully whether such a design should be a part of future studies.

Whole-herd anthelmintic treatment of dairy cows is commonplace and is likely to increase the development of anthelmintic resistance. This topic remains important for animal health, production efficiency, and maintenance of product efficacy into the future.
